# The Rift Valley Fever virus protein NSm and putative cellular protein interactions

**DOI:** 10.1186/1743-422X-9-139

**Published:** 2012-07-28

**Authors:** Cecilia Engdahl, Jonas Näslund, Lena Lindgren, Clas Ahlm, Göran Bucht

**Affiliations:** 1 Department of Clinical Microbiology, Umeå University, SE-90187, Umeå, Sweden; 2 Swedish Defence Research Agency, SE-90182, Umeå, Sweden

**Keywords:** Rift Valley fever, NSm, Yeast two-hybrid, SNAP-25, Ppil2, Cpsf2

## Abstract

Rift Valley Fever is an infectious viral disease and an emerging problem in many countries of Africa and on the Arabian Peninsula. The causative virus is predominantly transmitted by mosquitoes and high mortality and abortion rates characterize outbreaks in animals while symptoms ranging from mild to life-threatening encephalitis and hemorrhagic fever are noticed among infected humans. For a better prevention and treatment of the infection, an increased knowledge of the infectious process of the virus is required.

The focus of this work was to identify protein-protein interactions between the non-structural protein (NSm), encoded by the M-segment of the virus, and host cell proteins. This study was initiated by screening approximately 26 million cDNA clones of a mouse embryonic cDNA library for interactions with the NSm protein using a yeast two-hybrid system.

We have identified nine murine proteins that interact with NSm protein of Rift Valley Fever virus, and the putative protein-protein interactions were confirmed by growth selection procedures and β-gal activity measurements. Our results suggest that the cleavage and polyadenylation specificity factor subunit 2 (Cpsf2), the peptidyl-prolyl cis-trans isomerase (cyclophilin)-like 2 protein (Ppil2), and the synaptosome-associated protein of 25 kDa (SNAP-25) are the most promising targets for the NSm protein of the virus during an infection.

## Background

Rift Valley Fever (RVF) is classified as an emerging infectious disease, first identified in Rift Valley of Kenya in 1930 [1]. RVF was later reported throughout the sub-Saharan countries and Egypt in 1977–78. By the year 2000 the virus had spread to countries outside the African continent and for the first time was observed in Saudi Arabia and Yemen on the Arabian Peninsula [2,3]. Outbreaks caused by Rift Valley Fever virus (RVFV) results in serious socio-economic and public health impact for endemic communities of the African continent and the Arabian Peninsula.

Rift Valley fever is a reoccurring mosquito-borne viral disease able to cause abortions, fetal malformations and sudden deaths among ruminants; most susceptible are calves, lambs and kids [4-7]. Humans may get infected by the bite of an infected mosquito, by direct contact with infected animals or through body fluids or tissues thereof [8,9]. Symptoms with various severity are observed in humans ranging from influenza-like symptoms to retinitis, encephalitis and/or hemorrhagic fever which may result in a fatal outcome [2,6,10-12]. RVFV is believed to be transmitted by several mosquito species to a wide range of susceptible animals and outbreaks of RVF are linked to rainfall and flooding’s that provide for better breeding conditions of the mosquitoes [13].

Several vaccines and vaccine candidates have been developed for livestock and personnel with an occupational risk for the disease, but none are yet approved for public use outside endemic areas [14,15]. Safe vaccines applicable for animals and humans are therefore highly needed to prevent the spread of RVFV. For that purpose, a deeper understanding of the various functions of viral proteins, in combination with a more comprehensive understanding of the infectious process is required.

The RVF virion (*Bunyaviridae* family, *phlebovirus* genus) is spherical, 80–120 nm in diameter and coated by a bi-lipid envelope containing two viral glycoproteins; Gn and Gc. The virus has a three-segmented single-stranded RNA genome consisting of small (S), medium (M) and large (L) RNA segments of negative polarity ( Additional file 1: Figure S1) [8,16,17]. The L-segment encodes the multifunctional RNA-dependent RNA polymerase (RdRp). The S-segment codes for the structural nucleocapsid protein (N), that encapsulates the RNA molecules, and the non-structural protein (NSs) in an ambisense manner. The NSs protein suppresses the host immune response during an infection and is considered as the major virulence factor [18-22]. The M segment encodes two envelope glycoproteins, Gn and Gc, responsible for recognizing host cell receptors, and a non-structural protein (NSm) with basically unknown function. The M segment is transcribed into one transcript and the resulting polyprotein is subsequently cleaved into the above protein products. The NSm protein is obtained when translation starts from the second initiation codons ( Additional file 1: Figure S1).

The fact that the evolutionary preserved NSm is dispensable for virus replication in cell lines is interesting and indicates an involvement *in vivo*[23,24]. This suggestion is supported by the finding that RVFV recombinants lacking NSm show reduced virulence in rodents [25]. Other observations suggest that NSm is involved in the suppression of apoptosis [26] probably by activating the p38 MAPK cascade [27].

The main focus of this work was to identify protein-protein interactions between the NSm protein of RVFV and host cell proteins, with an intention to decipher the functions of this protein during the infectious process. Our results suggest nine putative cellular proteins able to interact with the NSm protein of RVFV; of these the Cpsf2, Ppil2 and SNAP-25 show the strongest interactions when using a yeast two-hybrid system.

## Results

### Cloning of *NSm* gene and screening the mouse-embryonic cDNA library

**Figure 1:**
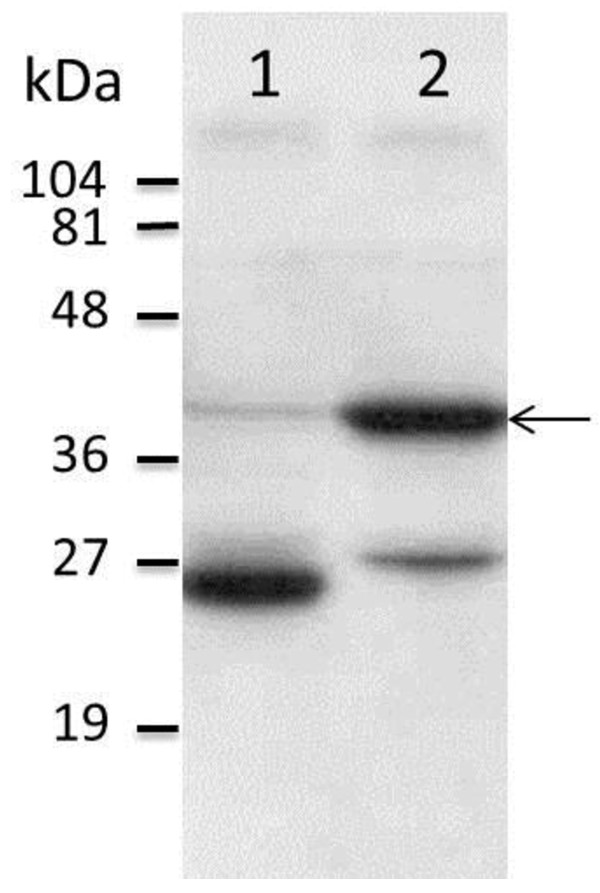
**Western blot showing protein expression of NSm.** Figure 1 demonstrates a Western blot analysis of proteins expressed by yeast cells. The individual proteins were separated by SDS-PAGE and visualized by c-Myc monoclonal antibodies. Lane 1 shows the pGBKT7-”empty vector” control, (GAL4 DNA binding domain protein in fusion with the c-Myc epitope tag). Lane 2 shows the fusion protein expressed from construct pGBKT7-NSm (the NSm protein in fusion with the GAL4 DNA binding domain protein and c-Myc epitope tag). The arrow adjacent to lane 2 indicates a protein band corresponding to the size of the fusion protein. A molecular weight standard is shown next to the figure.

The *NSm* gene of RVFV was amplified by PCR and the resulting product was ligated into the pGBKT7 vector, in fusion with the GAL4 DNA binding domain (BD). Correct clones were verified by DNA sequencing and protein expression in a yeast cell system resulted in a distinct band of the expected size (GAL4 DNA BD and c-Myc epitope tag < 27 kDa + NSm 14 kDa), as identified by SDS-PAGE followed by Western blot analysis (Figure 1).

The initial library screen resulted in 18 open reading frames (ORF’s), although the subsequent sequence analysis only identified nine different proteins (Table 1). Interestingly, eight of the 18 clones encoded the ORF of the same protein; the synaptosome-associated protein of 25 kDa (SNAP-25). Furthermore, the ring finger and CHY zinc finger domain-containing protein 1 / p53-Inducible Ring-H2 protein (Rchy1/Pirh2) and the serine/arginine-rich splicing factor 3 (Srsf3) was represented twice while the remaining six proteins were found as single cDNA clones.

**Table 1:** Summary of the cDNA encoded proteins

**Gene**	**Definition**	**Size (bp) / Complete ORF**	**Number of clones**	Interaction on solid media*	β-gal activity**
** *SNAP-25* **	Synaptosome-associated protein of 25 kDa	411 / Yes	8	+	70%
** *RCHY1 / Pirh2* **	Ring finger and CHY zinc finger domain-containing protein 1 / p53-Inducible Ring-H2 protein	786 / Yes	2	++	10%
** *Srsf3* **	Serine/arginine-rich splicing factor 3	495 / No	2	++	0%
** *Ppil2* **	peptidylprolyl isomerase (cyclophilin)-like 2	1566 / No	1	++	150%
** *Ewsr1* **	Ewing sarcoma breakpoint region 1	1968 / Yes	1	++	10%
** *St13* **	Suppression of tumorigenicity 13	1116 / Yes	1	+	0%
** *ND1* **	NADH dehydrogenase subunit 1	954 / No	1	++	50%
** *Cpsf2* **	Cleavage and polyadenylation specificity factor, subunit 2	2346 / No	1	++	350%
** *Efcab7* **	EF-hand calcium-binding domain-containing protein 7	1884 / No	1	+	50%

*Results from growth on selective solid media experiments are presented as; (+), growth at 30°C; (++), growth also at 37°C.

**Result from the β-galalactosidase activity measurements are given as Miller units in percentage to the positive control that was set to 100%.

### Growth on selective solid media

**Figure 2:**
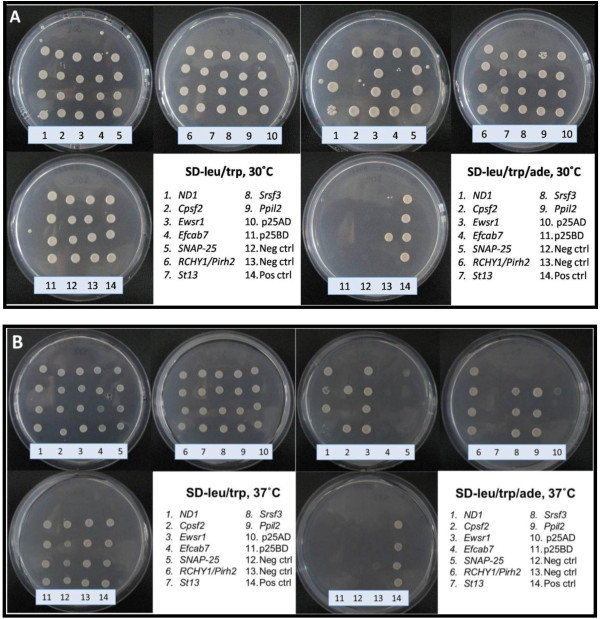
**Yeast growth on selective solid media demonstrating NSm interaction with cellular proteins.** Figure 2 shows photographs of agar plates demonstrating the growth of individual yeast clones on control plates (SD-leu/trp) and selective solid media (SD-leu/trp/ade) at optimal temperature (**A**) and elevated temperature (**B**). The genes encoded by the individual clones are listed within the figure. This figure show one of three individual repeats of this experiment and quadruplets of each yeast construct were tested at each time point.

To evaluate the interactions between the NSm protein and proteins from the initial screen, the yeast strain *S. cerevisiae* AH109 was pairwise transformed with one of the 18 cDNA clones and plasmid DNA expressing the NSm protein. Accurate interactions are expected to activate transcription of the *ADE2* reporter gene and thus allow growth on defined minimal media devoid of adenine. All 18 ORF’s, when co-transformed with the NSm construct, grew well on synthetic defined (SD) solid media (SD-leu/trp/ade) at 30°C, strengthening our previous observations from the library screen (Figure 2A). Furthermore, yeast cells expressing six of the nine identified proteins were also able to grow at high stringency (37°C) (Figure 2B). However, yeast clones expressing SNAP-25, St13 and Efcab7 showed weaker growth at elevated temperature, compared to growth on positive control plates (SD-leu/trp). As negative controls, individual transformation of the plasmids into AH109 did not result in *ADE2* gene activation and consequently no growth on selective solid media at the above temperatures (data not shown).

### ß-galactosidase activity in liquid cultures

**Figure 3:**
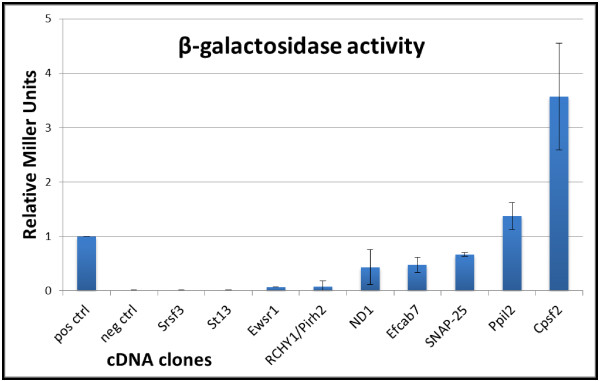
**ß-gal activity as an indicator of protein-protein interaction strength.** Figure 3 shows a graphical view of the relative strength of the protein-protein interaction based on β-gal activity measurements between NSm protein and indicated proteins. The strength of the interactions are expressed in Miller units and normalised to the positive control. The presented data and standard deviations are based on four individual experiments and duplicate samples.

The strength of the interactions between RVFV NSm protein and proteins identified by the library screen was investigated by measuring the ß-galactosidase (ß-gal) activity in liquid cultures. Of the 18 clones investigated two proteins; Cpsf2 and Ppil2 induced significantly higher ß-gal activity than the positive control (Figure 3). The relative induction in Miller units was calculated to 3.5 and 1.5, respectively. These results are in agreement with the above results from growth on solid media. Furthermore, one of the SNAP-25 clones, together with ND1 and Efcab7, also showed significant interaction with NSm (relative Miller units of 0.7, 0.5 and 0.5 respectively), in contrast to the other examined proteins and negative control samples as shown in Figure 3.

## Discussion

Outbreaks caused by Rift Valley Fever virus are devastating for affected communities and endemic regions of the developing world. In the efforts to develop prevention and efficient medical treatment against RVF, a comprehensive understanding of the infectious process, in combination with a deeper understanding of the molecular functions and relations between viral and host cell proteins, are of outmost importance.

The aim of the study was to identify murine proteins able to interact with the NSm protein of RVFV. For that purpose, an initial yeast mating procedure and library screen of a mouse embryonic cDNA library was conducted that resulted in 18 potential protein interactions. Among the positive clones nine different proteins were identified. The gene products, encoded by the identified clones, were then investigated for their ability to restore growth on selective growth media (SD – leu/trp/ade), as an indicator of reporter gene *(ADE2)* activation. At 30°C, all 18 clones grew well when co-expressed with NSm, supporting the results from the library screen. The same set of clones was also analyzed for growth at higher stringency (37°C); to select for strong protein-protein interactions. At this elevated temperature, six of the 18 clones were able to grow, in addition to the positive control. Consequently, these proteins (Ewsr1, RCHY1/Pirh2, Srsf3, ND1, Cpsf2 and Ppil2) became the most interesting proteins for further analysis.

Unexpectedly, sequence analysis of the 18 cDNA clones concluded that eight of them covered the ORF of the same protein; SNAP-25 (the synaptosome-associated protein of 25 kDa). SNAP-25 belongs to a family of highly conserved SNARE proteins with a cellular role to mediate vesicle docking, fusion and exocytosis by bringing adjacent cell membranes into close proximity and allowing membrane fusion [28]. SNAP-25 is a cytosolic, membrane-anchored protein that, together with syntaxin and synaptobrevin/VAMP, forms the synaptic SNARE-complex, responsible for Ca^2+^-dependent neurotransmission [29]. Expression of SNAP-25 is thus located to neuronal cells [30] mainly located in the brain [31]. Using a rodent model, the accumulation of RVFV to the liver indicates this organ to be a major replication site, but increased viral-levels are also found in the brain [32,33]. RVFV invasion to the central nervous system (CNS) may lead to several neurological symptoms, commonly observed at later stages of RVF virus infection [34,35]. SNAP-25 has several homologues represented in different tissue types [31]. One example is SNAP-23 which show 72% similarity to SNAP-25, and has been found to regulate secretion of cytokines from immune cells [36].

The strength of the protein-protein interactions was analyzed using *lacZ* as the reporter gene and the obtained ß-galactosidase activity was used as a semiquantitative indicator for the strength of the interaction, in comparison with the results of the positive control. However, only one of the eight SNAP-25 clones showed high β-gal levels and this specific clone was chosen for further experiments. Although the Ewsr1, RCHY1/Pirh2 and the Srsf3 proteins also showed positive interactions with the NSm protein by growth experiment on selective solid media, but their β-gal levels were low and these proteins were therefore excluded from this study.

Ppil2 (the peptidyl-prolyl cis-trans isomerase (cyclophilin)-like 2), also called the cyclophilin 60 (Cyp60) protein, induced significant ß-galactosidase activity. This interesting protein is a member of the cyclophilin family of peptidyl-prolyl isomerases, which is a group of highly conserved ubiquitous proteins [37]. Cyclophilins are involved in catalyzing the cis-trans isomerization reaction of proline, a rate-limiting step of protein folding by acting as chaperones in intracellular protein trafficking. The active site of this protein is the target for the immunosuppressor cyclosporine A (CyA) [38]. CyA is a drug used to suppress rejection of donor organs, and the Cyclophilin/CyA- complex inhibit transcription of several important genes in T-cells and thereby suppresses the immune response [39]. Ppil2 have recently also been shown to regulate cell surface expression of CD147 [38], a widely expressed integral plasma membrane glycoprotein. CD147 is responsible for a variety of activities including the role as a receptor for extracellular cyclophilins [40]. The extracellular cyclophilin-CD147 interactions seem to regulate chemotactic responses in many physiological and pathological processes including cell-mediated immunity and inflammation [38,41].

The strongest interaction, as indicated by the ß-galactosidase activity, was obtained when using Cpsf2 (the cleavage and polyadenylation specificity factor subunit 2) as a protein partner for NSm. The Cpsf2 protein is a component of a multi-protein complex that, together with the poly (A) polymerase and several other factors, carry out pre-mRNA 3’-end processing and formation [42] and hence play an important role in nuclear export, translational initiation and transcript stability [43]. Several viral infections are associated with defects in the mechanism of 3’-end processing and polyadenylation of mRNA, for example Influenza A virus and HIV. But, these viruses interact with other subunits of the Cpsf-protein complex [44]. Transcripts of RVFV are not polyadenylated at their 3’ ends, indicating that if this interaction plays a role during an infection it may affect the infection indirectly by disturbing the processing of host cell mRNAs.

A noted problem with the yeast two-hybrid screens is the potential risk of getting false positive results. The fact that many of the positive clones obtained from the library screen encode the same protein, and the indicated proteins grow at low and high stringent conditions, further supports our findings. Finally, the clones considered positive showed more than 30 times higher ß-gal activity than the negative control.

We believe that the results presented in this study are of high importance since this is the first report to suggest mammalian protein partners for the obscure NSm protein. New knowledge regarding NSm is necessary for a more comprehensive understanding of the role of the NSm protein during the infectious process and for the ongoing search for prevention and treatment of Rift Valley fever. Ongoing experiments include tagging these proteins pair-wise with different epitopes for monoclonal antibodies. Suggested interactions may then be proven by the detection of the corresponding protein partner by pull-down experiments.

Since the continuation of these results may be addressed in a number of different ways, we hope that our results will inspire other groups to address these questions by different approaches.

## Conclusion

It has been difficult to find a role for the RVFV NSm even though the coding sequence of this protein has been preserved over time. The knowledge that NSm is not essential for growth in cultured mammalian cells might suggest that this protein may have roles in pathogenesis and future experiments using animal or mosquito models systems may answer this question. Moreover, we cannot rule out a function for this protein for infection of insect cells.

Our findings suggest several possible protein-protein interactions. The three most interesting proteins from our study are Cpsf2, Ppil2 and SNAP-25. These proteins are all involved in fundamental biological pathways in the eukaryotic cell and could be disturbed if targeted by a viral protein.

## Methods

### Yeast culture and transformation

Growth and transformation of *Saccharomyces cerevisiae* yeast cells were basically performed according to the Yeast Protocol Handbook (Clontech Laboratories, Inc., Mountain View, CA, USA). Prior to transformation, fresh (not older than 4–5 days) individual colonies of yeast were inoculated in 10 ml liquid YPD medium (10 g yeast extract, 20 g Difco peptone, 1 L H_2_O, pH: 5.8) supplemented with L-adenine hemisulfate (20 mg/L), glucose (2%) and incubated o/n at 30°C with shaking (250 rpm). The culture was thereafter diluted with approximately 90 ml fresh medium to an OD_600_ of 0.2-0.3 and grown to OD_600_ 0.5-0.6 before harvested by centrifugation. The obtained samples were washed once by centrifugation and resuspended in 3 ml 1xTE/LiOAc (0.01 M Tris–HCl, 1 M EDTA, pH: 7.5 / 0.1 M LiOAc, pH: 7.5). Prior to transformation, 150 μl of the yeast cells were added to a mixture of 0.1 mg herring sperm carrier DNA (denatured for 10 min at 95°C) and 0.1 mg of each DNA plasmid. The samples were mixed thoroughly for one min and 700 μl 1xTE/LiOAc/PEG (40%) were added. The samples were then incubated for 30 min at 30°C with slow shaking (75 rpm) followed by a 42°C heat shock for 20 min. The yeast cells were subsequently collected by centrifugation and the pellets were washed once and resuspended in 150 μl of H_2_O. The transformation mixture was finally spread on selective solid agar media, SD-leu/trp (to select for both plasmids) and incubated at 30°C for 3–4 days.

### Cloning of the *NSm* gene

The *NSm* gene from the RVFV strain ZH548 [45] was amplified by PCR from the second methionine residue of the M-segment, using the forward: 5’ - CATGGAGGCCGAATTCATGATTGAAGGAGCTTGGG – 3’ and reverse: 5’ – GGATCCCCGGGAATTCAGCAAAAACAACAGGTGCC – 3’ primers. The primers were designed to extend the 5’ and 3’ ends of the *NSm* gene with approximately 15 nt that are homologous to the flanking regions of the EcoRI digested pGBKT7 vector. Prior to transformation, the PCR product was ligated using the In-Fusion™ PCR Cloning System (Clontech Laboratories, Inc., Mountain View, CA, USA) into the linearized vector, downstream the DNA-BD, the ATG and the c-Myc epitope tag encoded by the vector. Correct constructs were identified in recombinants of *E. coli* by PCR screening and DNA sequencing (Eurofins MWG Operon, Ebersberg, Germany). Plasmid DNA of correct clones were prepared from cultures of *E.coli* and transformed into the yeast strain AH109 using the above procedures (Table 2). A protein of the correct size was identified by sodium dodecyl sulfate polyacrylamide gel electrophoresis (SDS-PAGE) followed by Western blot analysis. Briefly, the Western blot analysis was performed after transfer of proteins from SDS-PAGE gels to Hybond-N membranes (Amersham Pharmacia Biotech Inc.) and antibodies directed against the c-Myc epitope of the tagged fusion protein (NSm + GAL4 DNA-BD) (c-Myc monoclonal antibody). Secondary goat-anti-mouse-peroxidase labeled antibodies (Clontech Laboratories Inc.) and the enhanced chemiluminescence (ECL+) kit (Amersham Biosciences) was used to visualize the correct band.

**Table 2:** Strains and plasmids used in this study

**Yeast strain**	**Genotype**		
	*MATα, ura3-52, his3-200, ade2-101, trp1-901, leu2-3,*		
Y187	*112, gal4Δ, met*^*-*^*, gal80Δ, URA3::GAL1*_*UAS*_*-GAL1*_*TATA*_*-lacZ, MEL1*		
	*MATa, ura3-52, his3-200, trp1-901, leu2-3,*		
AH109	*112, gal4Δ, gal80Δ, LYS2::GAL1*_*UAS*_*-GAL1*_*TATA*_*-HIS3, MEL1*		
	*GAL2*_*UAS*_*-GAL2*_*TATA*_*-ADE2, URA3::MEL1*_*UAS*_*-MEL1*_*TATA*_*-lacZ*		
**Plasmid**	**Size (kb)**	**Selectable marker on SD-media**	**Additional information**
pGADT7	8.0	-Leu	*GAL4*_(768–881)_AD, *LEU2*, amp^r^, HA epitope tag
pGBKT7	7.3	-Trp	*GAL4*_(1–147)_DNA-BD, *TRP1,* kan^r^, c-Myc epitope tag

### Yeast mating library screen

Screening procedures of a cDNA library containing approximately 26 million clones (Matchmaker 17 day mEmbryo in Yeast, Clontech Laboratories, Inc.) were done using the yeast mating method according to manufacturer’s instructions. Briefly; fresh colonies of two mating strains; Y187 (holding the cDNA library) and AH109, (holding the *NSm* gene) were incubated o/n at 30°C in 0.5 ml rich YPD medium. One hundred μl aliquots of the mating culture were thereafter spread on solid SD-leu/trp/ade/his agar plates to select for both plasmids.

### Bioinformatics

The plasmids encoding putative protein-protein partners to NSm, were purified according to manufacturer’s instructions using spin Miniprep kit columns (Qiagen Inc., Hilden, Germany) and the inserts were sequenced by Eurofins MWG Operon, Ebersberg, Germany with T7 and AD sequencing primers, as suggested by the pGADT7AD information data sheet (Clontech Laboratories, Inc., Mountain View, CA, USA). The obtained sequences were finally analysed by using SeqMan Pro; Version 8.1.5(3), 414 (Lasergene, DNASTAR™) and the resulting gene sequences were analysed against the NCBI GenBank database to determine the identity of the encoded gene products.

### Plate selection assay

The protein-protein interactions between the NSm protein and clones of the library were investigated by growth on SD media through a selection procedure using the principle of yeast two-hybrid system. In summary, the *S. cerevisiae* reporter strain AH109 was co-transformed with the plasmid pGADT7 expressing the GAL4 activating domain (AD), fused to proteins of the initial library screen, and the GAL4 DNA-BD plasmid pGBKT7 fused to the RVF *NSm* gene (Table 2). After four days of growth on SD-leu/trp plates, four fresh colonies were picked and resuspended in 50 μl of sterile water. Aliquots of 5 μl were thereafter spotted on two sets of replica plates; each set containing one SD-leu/trp (control plate) and one SD-leu/trp/ade (selective media) and incubated for another four days. The first set of plates was incubated at 30°C while the second set was kept at 37°C. Growth on the selective media (SD-leu/trp/ade) indicates that protein-protein interaction has occurred through induced transcription of the *ADE2* reporter gene. The constructs pGADT7 carrying *F. tularensis IglA* and pGBKT7 carrying *F. tularensis IglB* (kindly provided by Jeanette Bröms) was used as positive control [46]. *S. cerevisiae* strain AH109 co-transformed with either pGADT7:*IglA* and pGBKT7:*NSm* or pGADT7:*NSm* and pGBKT7:*IglB* were used as negative controls.

### ß-galactosidase assay

The strength of the protein interactions towards the NSm protein were also investigated by measuring the ß-galactosidase (ß-gal) activity in liquid cultures. Briefly, the *S. cerevisiae* reporter strain Y187 was co-transformed, as described above for AH109 and yeast cells, and grown in 5 ml selective SD-leu/trp medium at 30°C (Table 2). Overnight cultures were diluted in 6 ml rich YPD-medium to OD_600_ of 0.2-0.3 and aliquots of 1.5 ml of each culture were harvested at an OD_600_ of 0.5-0.8, by centrifugation, washed once and resuspended in 300 μl Z-buffer (16.1 g Na_2_HPO_4_*7H_2_O, 5.5 g NaH_2_PO_4_*H_2_O, 0.75 g KCl, 0.246 g MgSO_4_*7H_2_O, 1 L H_2_O, pH: 7.0). Samples of 100 μl were lysed by three cycles of freeze-thawing in liquid nitrogen and 37°C water bath, before the enzyme activity was analysed using Z-buffer containing 0.27% ß-mercaptoethanol and 4 mg/ml of ONPG (ortho-nitrophenol-ß-D-galactopyranoside) as substrate. The reaction mixture was incubated at 30°C before stopped at different time points by the addition of 400 μl Na_2_CO_3_ (1 M). Cell debris was removed by centrifugation for 10 min at 14 000 rpm and OD_420_-values were recorded. The expressed enzyme activity was calculated in Miller units as described earlier [47] and normalised to the positive control (100%).

## Competing interests

The authors declare that they have no competing interests.

## Authors’ contributions

CE and JN performed the practical work, did the analyses and contributed to writing of the manuscript. LL performed the screening of the library and cloning of the NSm gene. CA participated in coordination and contributed to the writing and proof reading. GB participated in the study design, coordination and drafting of the manuscript. All authors read and approved the final manuscript.

## Supplementary Material

Additional file 1 **Figure S1. A schematic figure of the three-segmented RVFV genome.** Additional file 1: Figure S1 illustrates the three-segmented RVFV genome, the size of the three RNA segments and the encoded proteins. The upper part of the figure shows the S segment and the two genes encoding the N and NSs protein with the inter-genomic region separating the two coding sequences. The middle part of this figure demonstrates the L segment and the multifunctional RNA dependent RNA polymerase (RdRp) protein encoded in an antisense manner. The cartoon at the bottom of the figure shows the M segment and the polyprotein precursor that is subsequently cleaved into the NSm, Gn and Gc proteins. The five potential in frame translation initiation codons are shown below in a magnified picture of the NSm encoding region. The F and the R primers used to amplify the NSm gene, from the proposed second AUG codon, are also shown. (PPTX 119 kb) Click here for file
