# Supplementary material for: The Rift Valley Fever virus protein NSm and putative cellular protein interactions
Source: Virol J. 2012 Jul 28;9:139. doi: 10.1186/1743-422X-9-139 (PMC3439357; doi:10.1186/1743-422X-9-139)
Supplement: Additional file 1 — Figure S1. A schematic figure of the three-segmented RVFV genome. Additional file 1: Figure S1 illustrates the three-segmented RVFV genome, the size of the three RNA segments and the encoded proteins. The upper part of the figure shows the S segment and the two genes encoding the N and NSs protein with the inter-genomic region separating the two coding sequences. The middle part of this figure demonstrates the L segment and the multifunctional RNA dependent RNA polymerase (RdRp) protein encoded in an antisense manner. The cartoon at the bottom of the figure shows the M segment and the polyprotein precursor that is subsequently cleaved into the NSm, Gn and Gc proteins. The five potential in frame translation initiation codons are shown below in a magnified picture of the NSm encoding region. The F and the R primers used to amplify the NSm gene, from the proposed second AUG codon, are also shown. (PPTX 119 kb) [file 1743-422X-9-139-S1.pptx]

## Slide 1
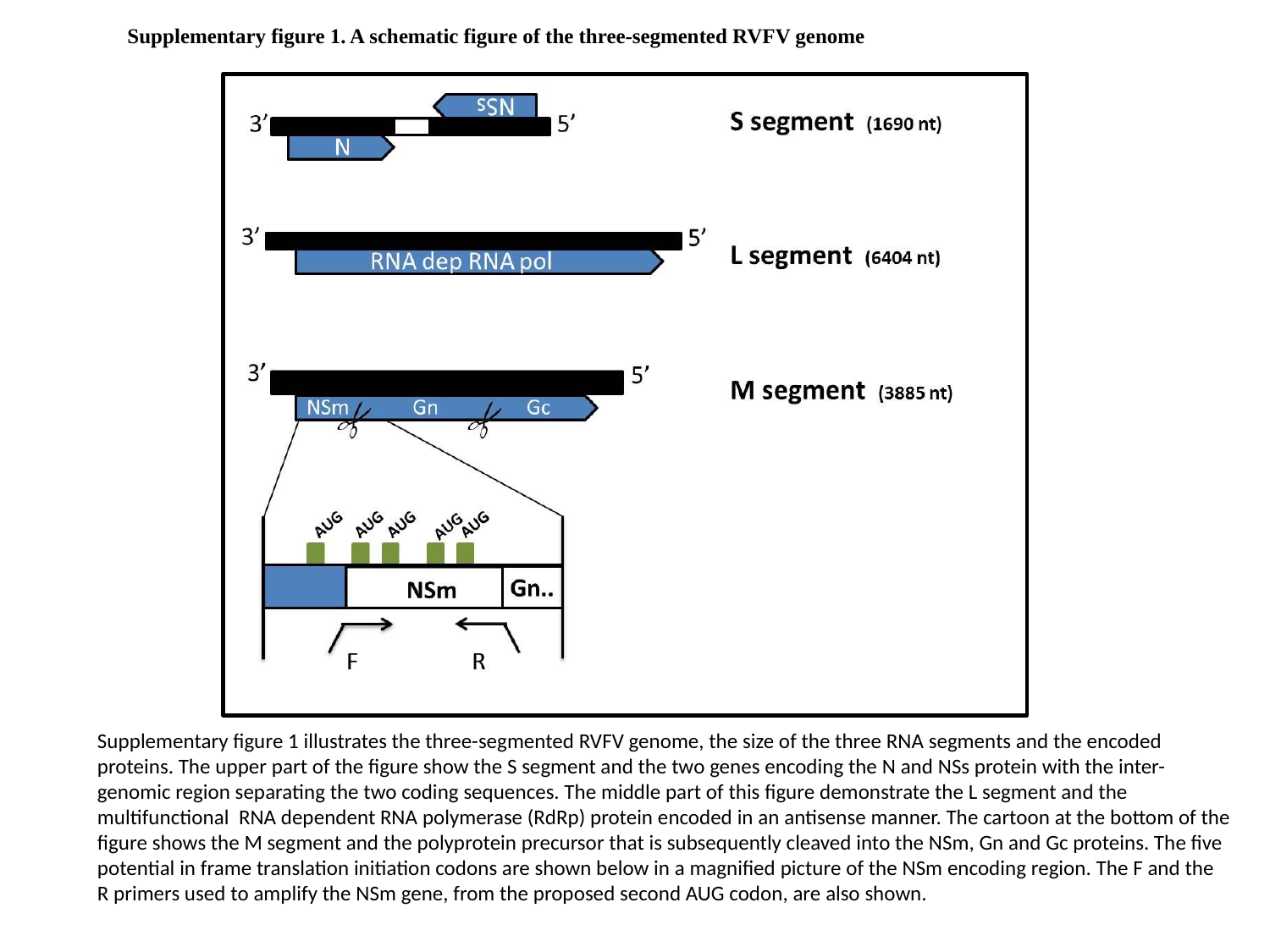

Supplementary figure 1. A schematic figure of the three-segmented RVFV genome
Supplementary figure 1 illustrates the three-segmented RVFV genome, the size of the three RNA segments and the encoded proteins. The upper part of the figure show the S segment and the two genes encoding the N and NSs protein with the inter-genomic region separating the two coding sequences. The middle part of this figure demonstrate the L segment and the multifunctional RNA dependent RNA polymerase (RdRp) protein encoded in an antisense manner. The cartoon at the bottom of the figure shows the M segment and the polyprotein precursor that is subsequently cleaved into the NSm, Gn and Gc proteins. The five potential in frame translation initiation codons are shown below in a magnified picture of the NSm encoding region. The F and the R primers used to amplify the NSm gene, from the proposed second AUG codon, are also shown.
